# MIC-O-MAP: a technology-enhanced learning environment for developing micro-macro thinking skills

**DOI:** 10.1186/s41039-017-0063-7

**Published:** 2017-12-06

**Authors:** Anura B. Kenkre, Sahana Murthy

**Affiliations:** Inter-Disciplinary Program in Educational Technology, Indian Institute of Technology, Bombay, India

**Keywords:** Micro-macro thinking, TEL environment, Design-based research

## Abstract

MIC-O-MAP (MICroscopic-Observations-Macroscopic-Predictions) is a technology-enhanced learning (TEL) environment designed for developing micro-macro thinking skills among science and engineering undergraduate students. Micro-macro thinking involves being able to analyze dynamic processes and interactions on a microscopic level and establish co-relations to the outcomes which we can see and measure in the macroscopic world. In this paper, we report 2 cycles of iterative design, development, and evaluation of MIC-O-MAP, based on a design-based research approach. We first identify the pedagogical design features and learning activities of MIC-O-MAP based on a literature review of the development of micro-macro thinking. We then report an experimental study, which showed positive results that the design features and learning activities of MIC-O-MAP helped students develop micro-macro thinking. This was followed by a detailed interaction analysis, which provided insights into the redesign of MIC-O-MAP. An evaluation of the revised version of MIC-O-MAP showed that the shortcomings of the original version were addressed. The interaction analysis also led us to identify effective actions and learning paths as students learn in interactive TEL environments.

## Introduction

Science and engineering graduates are expected to be able to apply knowledge of mathematics, science, and engineering; formulate and solve complex problems; design and conduct experiments; and analyze and interpret data, design systems, and processes to meet desired needs (Shuman, Besterfield-Sacre & McGourty, 2005; Seehorn et al., 2011; Lawson, 1989). These process-related skills have been deemed to be important not only by educators but also by industries hiring science and engineering graduates (Lang et al., 1999). An effective way to facilitate the development of these skills is to engage students in tasks which require them to undertake the process of scientific inquiry within their undergraduate learning experience. Undertaking an inquiry-based learning task requires them to apply their theoretical knowledge to real-world situations. Particularly in science and engineering domains, such tasks involve relations between observable, tangible phenomena at a macroscopic level and their underlying structure, processes, and dynamics at a microscopic level.

Micro-macro thinking skill is developed when learners understand to use relations between observed phenomena at the macroscopic level and the models of invisible particles such as atoms or molecules at a microscopic level (Meijer, 2011). Learners need to describe, understand, and predict the outcomes of phenomena at the macroscopic level by relating these to the scientific models of structures and processes at the microscopic level. However, micro-macro thinking has been found to be problematic for students as they have difficulty in bridging the huge mental gap between microscopic and macroscopic levels (Harrison & Treagust, 2003; Eilam, 2004; Gilbert & Treagust, 2009; van Berkel et al., 2009). Hence, designing explicit instruction for developing micro-macro thinking skill is necessary.

Based on Meijer’s descriptions of microscopic and macroscopic levels (Meijer, 2011), we use the term “macro-world” to denote directly observable real-world phenomena and the term “micro-world” to refer to models with structures at the level of molecules or atoms. We define “micro-macro thinking” as the ability to establish a link between the variables in a micro-world and its corresponding manipulable variables in a macro-world in order to predict the functionality of the system. In a given situation, in order to establish this micro-macro link, sub-skills of making observations in the micro-world, predicting macroscopic outcomes, testing these outcomes against experimental evidence, and revising predictions (if necessary) need to be developed.

In order to address micro-macro thinking skills, many solutions have been employed in face-to-face settings as well as using technology-enhanced learning (TEL) environments. It is observed that these skills are developed to a limited extent in a traditional lecture-based classroom and to a large extent wherein face-to-face solutions such as Investigative Science Learning Environment (ISLE) (Etkina & Van Heuvelen, 2007) and Model-based Analysis and Reasoning in Science (MARS) (Raghavan & Glaser, 1995) get employed. Some examples of TEL environments that target these skills include Web-based Science Environment (WISE) (Slotta, 2004), Web-based inquirer with modeling and visualization technology (WiMVT) (Sun & Looi, 2013), Model-It (Fretz et al., 2002), Co-Labs (van Joolingen et al., 2005), and Go-Labs (Govaerts et al., 2013).

In this paper, we report the design, development, and evaluation of a TEL environment, MIC-O-MAP (MICroscopic-Observations-Macroscopic-Predictions), aimed to develop students’ micro-macro thinking in the context of self-regulated learning within tertiary education. MIC-O-MAP contains modules in the domain of basic electronics, which is commonly studied by students of many undergraduate science and engineering majors. Understanding of topics in this domain requires a firm understanding of both the micro-world (motion of electrons) and the macro-world (the tangible circuit and measurements used in the lab). MIC-O-MAP allows students to learn at their own pace along their own learning path. Its learning modules can be used by teachers as supplementary learning resources to traditional classroom instruction, or for independent learning.

We have followed a design-based research (DBR) approach with iterative cycles of design, development, and evaluation (Cobb et al., 2003). We report two DBR cycles which include the identification of the key features and learning activities of MIC-O-MAP, empirical studies using an experimental design, and a qualitative interaction analysis which provide insights into the elements of MIC-O-MAP that needed to be redesigned for effective learning. The key contributions of this research are as follows: (i) a detailed description of the features and learning activities of a TEL environment explicitly focused on the learners’ development of micro-macro thinking skill, tested for topics in the domain of analog electronics at the university level; (ii) an iterative process of design, evaluation, and redesign to develop TEL environments for specific learning goals; and (iii) identification of effective actions and learning paths as students interact with TEL environments.

## Literature review

### Micro-macro thinking skills

The practice of science and engineering requires that students develop science process skills, inquiry skills (de Jong, 2006), or scientific abilities (Etkina et al., 2006) in addition to content knowledge. Diverse research bodies such as ABET (Shuman, Besterfield-Sacre & McGourty, 2005), Washington Accord (Basri et al., 2004), and Next Generation Science Standards (NGSS) (Duschl & Bybee, 2014) have attempted defining a set of thinking skills expected from science and engineering graduates. The basis of many such process skills includes being able to observe and analyze dynamic processes and interactions on a microscopic level and establish co-relations to the outcomes which we can “see” and measure in the real world or a macroscopic world. The macroscopic level comprises the tangible and visible, and the microscopic level typically comprises an invisible particulate level, such as the electrons, molecules, or atoms (Johnstone, 1982). Research shows that students have difficulty in transferring from a macroscopic level of representation to the microscopic level (Gabel, 1998). It is important for instructors and curriculum designers to have in-depth knowledge of the problematic features of micro-macro thinking and understand what to be communicated to students and how to best communicate it to them (van Berkel, Pilot & Bulte, 2009).

These science and engineering process skills have been deemed important not only by educators but also by industries hiring science and engineering graduates (Lang et al., 1999). For example, electronics engineers designing a circuit need to understand not only the current-voltage relationships but also concepts of electron motion, band gap, barrier potential, etc., and how they affect the observed current-voltage graphs. Thinking skills related to establishing a micro-macro link in systems have been reported in various fields, for example by researchers focusing on helping students learn systems thinking in various domains in science and engineering (Wilensky & Resnick, 1999). A key aspect of systems thinking is the need to be able to think backward and forward between general systems models and concrete objects and processes. Similarly, educators who teach scientific modeling focus on explaining the macroscopic outcome or observed physical phenomena by applying a model at a microscopic level (Etkina, Warren & Gentile, 2006).

### TEL environments for developing micro-macro thinking skills

Researchers and educators have developed TEL environments that address one or more aspects of micro-macro thinking skills. These include WISE which provides an Internet-based platform for middle- and high-school science activities (Slotta, 2004) and MARS (Raghavan & Glaser, 1995) which is a semester-long science curriculum for middle-school students that was designed to foster the development of model-based reasoning skills. Methods such as ISLE (Etkina & Van Heuvelen, 2007) include classroom and lab activities and video-based experiments. WiMVT proposes an inquiry cycle incorporating eight phases: contextualize, question and hypothesize, pre-model, plan, investigate, model, reflect, and apply (Sun & Looi, 2013). Model-It argues that students go through an inquiry cycle in the following phases: planning, searching, synthesis, analysis, explaining, and evaluating (Fretz et al., 2002). Similar cycles are proposed by solutions such as Co-Lab (van Joolingen et al., 2005), Go-Lab (Govaerts et al., 2013), and Inquiry Island (White et al., 2002) which suggest usage of netbooks in addition to the above technology-based resources. These technology-based solutions have tremendous potential to provide visual representations of dynamic phenomena that involve change over time, and the power to make the invisible visible. Simulations provide the opportunity for stating and testing hypothesis and multiple representations of physical phenomena such as diagrams and graphs (Blake & Scanlon, 2007).

In all of these existing interventions, the teacher plays an important role by facilitating the process, giving prompts, or grading the student’s efforts at a later stage (Raghavan & Glaser, 1995). Many of these methods also include components such as lab work, field trips, discussions, and assignments apart from the TEL environment itself. Our solution is targeted towards creating a TEL environment in the context of self-regulated learning. It contains prompts and scaffolds to provide the learner guidance or help with troubleshooting during the learning process. This can be done at the pace of the learner. The time for all the activities is also not fixed, and students can repeat each task multiple times until the desired level of understanding is reached. In addition, most existing TEL environments described above are focused on middle- and high-school science curriculum. The goal of this paper is to design and evaluate a technology-enhanced learning environment which enables students to develop the ability of micro-macro thinking within the context of self-regulated learning in a tertiary education setting.

### Pedagogical underpinning in TEL environments

We review effective pedagogical methods and processes such as inquiry learning, self-regulated learning, scaffolding, and formative assessment and feedback.

Inquiry learning mimics authentic inquiry. Because they are closely related, they share the following constitutive cognitive processes (Quintana et al., 2004): orientation (identification of variables and relations), hypothesis generation (formulation of a statement or a set of statements, perhaps as a model), experimentation (changing variable values, making predictions, and interpreting outcomes), reaching conclusions (on the validity of the hypothesis), evaluation (reflection on the learning process and the acquired knowledge), planning (outlining a schedule for the inquiry process), and monitoring (maintaining an overview of the inquiry process and the developing knowledge) (De Jong, 2006). MIC-O-MAP has multiple features which allow students to undertake the inquiry cycle of observation-explanation-prediction-testing prediction. A simulation of the microscopic world allows the student to make observations, a prediction question feature where a macroscopic prediction has to be made based on the microscopic observations, a justification box wherein a micro-macro link needs to be established which can explain the choice of prediction, and a real-world answer in which the prediction can be tested.

Self-regulated learning is an active, constructive process whereby learners set goals for their learning and then attempt to monitor, regulate, and control their cognition, motivation, and behavior in the service of those goals (Winne, 2001). It is also viewed as proactive processes that students use to acquire academic skill and self-monitoring one’s effectiveness (Zimmerman, 2008; McLoughlin & Lee, 2010). SRL is guided and constrained both by personal characteristics of the learner and by contextual features of the environment (Pintrich, 2000). Thus, SRL models offer a comprehensive framework with which to examine how students learn and how they adapt during the learning process. In order to create an effective medium for self-regulated learning, designers should incorporate specific scaffolds in hypermedia environments designed to foster students’ conceptual understanding of complex topics (Azevedo, Cromley & Seibert 2004). Students’ use of effective strategies could be scaffolded within a technology-based environment by providing online prompts, possibly from an embedded animated pedagogical agent (Mayer, Dow, & Mayer, 2003), who provides instructions and feedback via a dialogue with the learner. It has also been suggested in a computer-based solution gStudy, to record traces of student methods of learning which can be later used to help struggling learners see which strategies work best for them (Winne et al., 2006). A note-taking function of this software has also been highlighted in order to help students in extracting key information or writing the summary of a section of the text (Zimmerman, 2008). These features have also been incorporated into MIC-O-MAP so as to aid students in tracing their path in order to reach their choice of feature or to write down the key observations/on-screen text provided in the form of instructions or feedback. Conceptual scaffolding questions with customized feedback have been included based on this theory so as to provide prompts towards key areas where more observations need to be made or hints towards establishing a micro-macro link. The current research points to a growing appreciation of the need to support and encourage the learner control over the whole/entire learning process (Dron, 2007).

Research in the area of formative assessment suggests the creation and administration of questions that can be used by students to assess their understanding of a topic or area of study (Black & Wiliam, 1998). Students find such questions useful to check their level of understanding. They often make repeated attempts at such questions in order to enhance their knowledge and skill acquisition. Effective feedback requires that students be informed how they perform in the light of what was attempted—actual versus ideal performance (Nicol & Macfarlane-Dick, 2006). Good quality external feedback is information that helps students troubleshoot their own performance and self-correct; that is, it helps the students take action to reduce the discrepancy between their intentions and the resulting effects (Butler & Winne, 1995). The conceptual scaffolding questions are continuously provided to students whenever they are unable to make a prediction or they require assistance in establishing or strengthening the micro-macro link.

## Design-based research method

DBR guides theory development, improves instructional design, extends the application of results, and identifies new design possibilities (Cobb et al., 2003; Edelson, 2002). A DBR approach consists of iterative cycles of design, enactment, analysis, and redesign. This helps in augmenting the intervention on the basis of the “failures” in the earlier research cycles. In order to get an idea about the extent to which features of the environment were able to achieve this goal, we embarked on an iterative cyclic process and adapted the DBR methodology by Reeves (2000). This process contains stages in which practical problems are analyzed by the researcher, a prototype of the solution is developed, iterative cycles of testing and refinement of solution are carried out, and design principles are generated based on reflection.

**Fig. 1:**
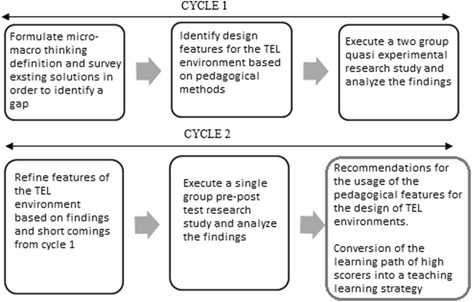
Design-based approach for developing our TEL environment

We adopted the DBR framework in our research work in an attempt towards identification of the features and learning activities required in a TEL environment for developing micro-macro thinking. Design-based research interventions are developed in a cyclic process of successive prototypes. We first conducted an exhaustive literature review in order to identify the features to be incorporated into the learning environment. Based on the experiments and the analysis of the data extracted from them, we refined and improvised the pedagogical features of our learning environment. Figure 1 depicts the process diagram which we have followed.

## Design of MIC-O-MAP: first cycle of DBR

### Overview of MIC-O-MAP learning design

We first identified the design features and learning activities for a TEL environment to develop micro-macro thinking based on the pedagogical underpinnings discussed in the “Pedagogical underpinning in TEL environments” section. The pedagogical goals of MIC-O-MAP are to identify objects, interactions, and processes in both micro- and macro-worlds individually, formalize these in terms of variables, and determine the relation between those variables. Students should then be able to link the micro- and macro-worlds by determining variables in the micro-world which are linked to macro variables and by describing the nature of the above relation. We call this relation “micro-macro link.” For applying this understanding, students should be able to predict outputs in macro-world given the dynamics or processes in micro-world. Micro-macro thinking skill is operationalized as a measureable set of abilities in which students learn to make predictions, test predictions with respect to experimental results, and revise predictions if necessary.

**Fig. 2:**
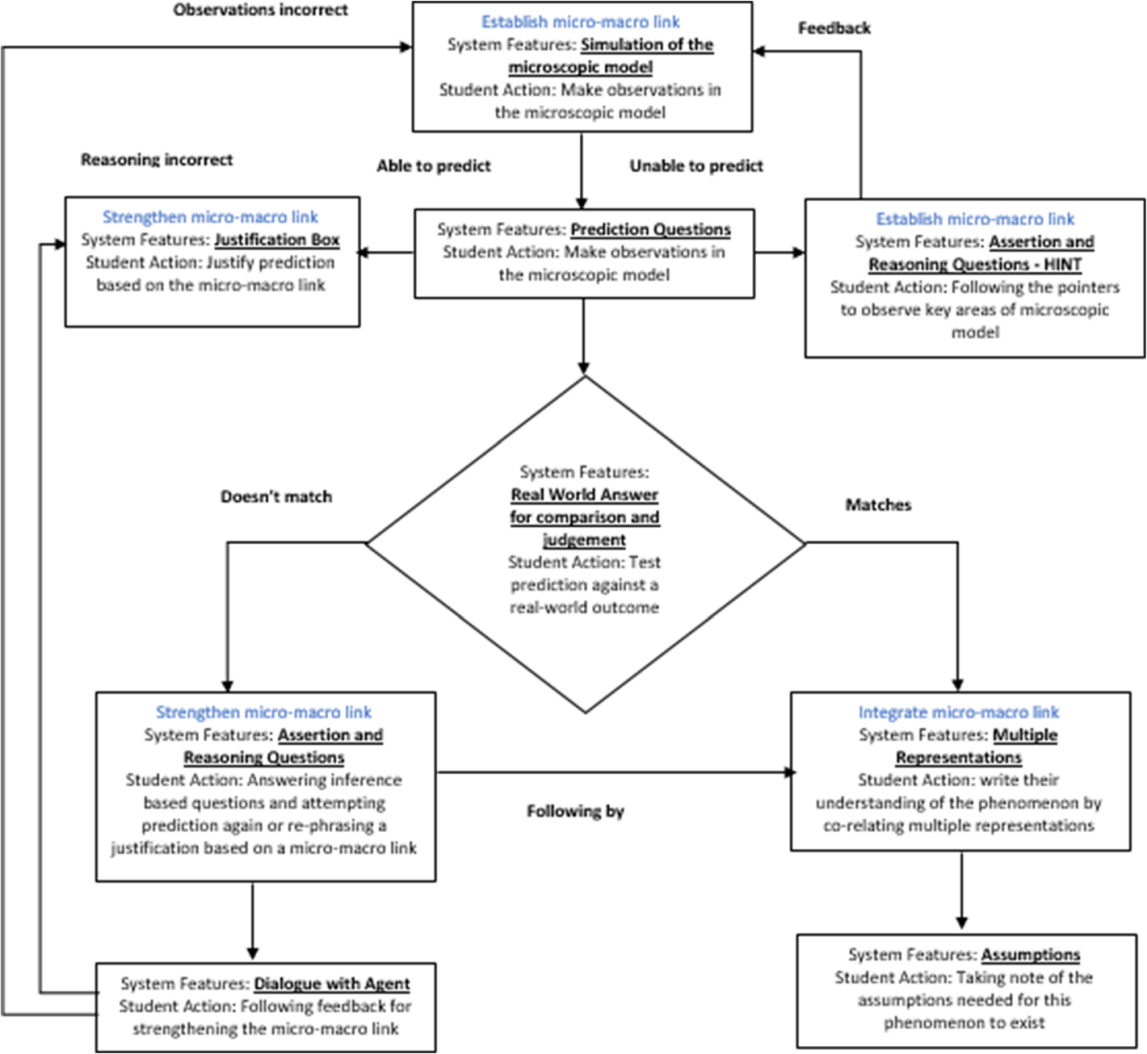
Overview of the TEL environment and local instructional theory

The local instructional theory (Cobb et al., 2003), i.e., description of a learning route based on a rationale for this learning environment, is elaborated in terms of three stepping stones: namely establishment of a micro-macro link, strengthening of the micro-macro link, and integration of the micro-macro link (Fig. 2).

- *Establishing micro-macro link*

**Fig. 3:**
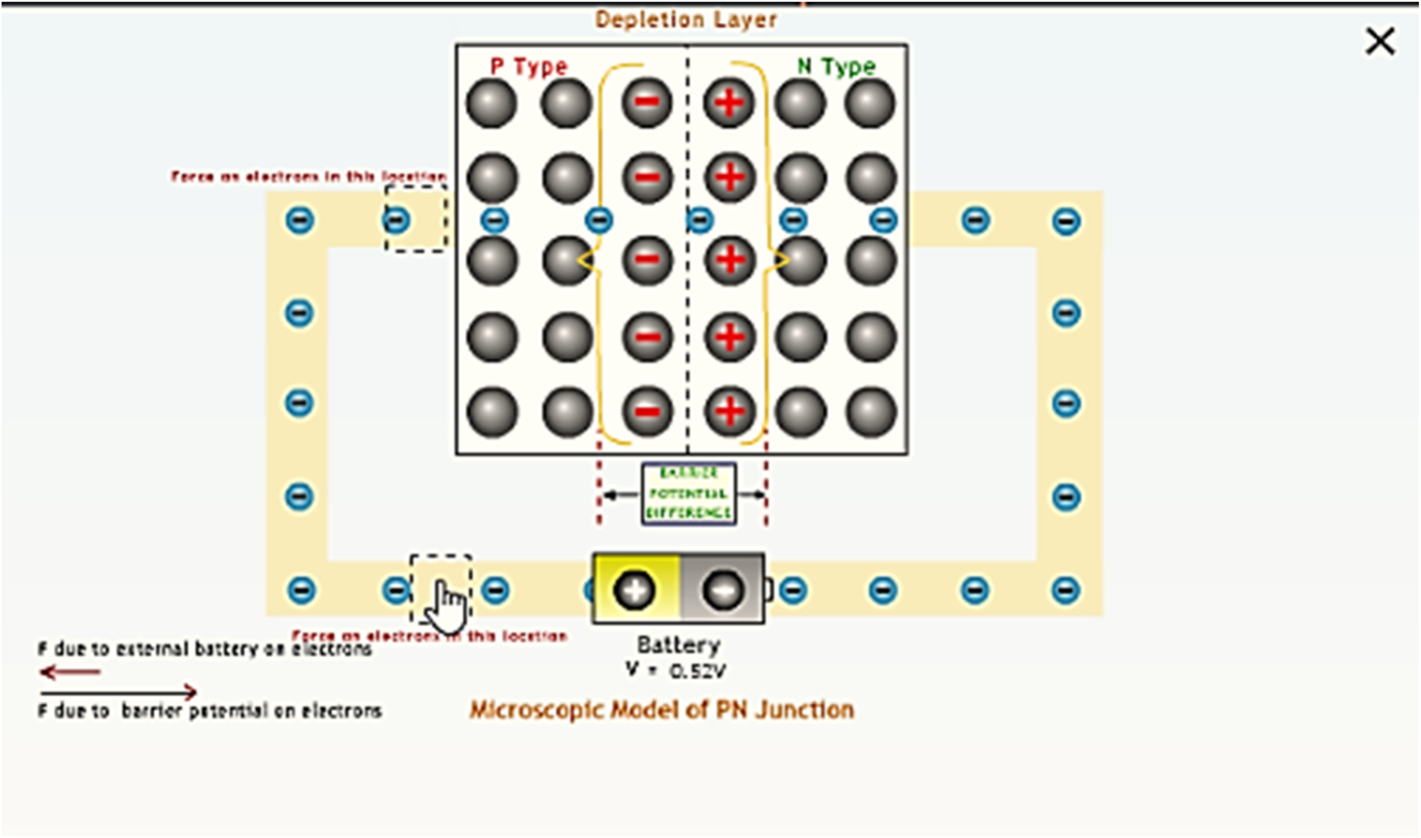
Simulation of the microscopic model

Micro-macro link is established when students are asked to make careful observations in a given simulation of the microscopic model and then use it to predict the macroscopic outcomes of the given physical phenomenon (Fig. 3). While doing this, students are able to establish a link between the occurrences and changes taking place at the microscopic level and its effect on the macroscopically observable physical quantities. They use this link and rationale while writing their justification behind a prediction made by them. An important aspect of scientific thinking is to explain the macroscopic outcome of a given situation by applying a model at a microscopic level. This helps students understand the mechanism underlying a phenomenon and can lead to the refinement of the conceptual understanding of the phenomenon (de Jong & van Joolingen, 1998).

- *Strengthening micro-macro link*

When students are unable to make a prediction or are falling short in establishing a micro-macro link, MIC-O-MAP provides them with a series of conflict resolution questions which aid in sense-making of the physical phenomenon being observed. There exists a guided enquiry beginning with the justification and reasoning provided by the student for making a particular choice of prediction. There onwards, they are asked multiple scaffolding questions along with customized feedback which either provides them with prompts for areas of the microscopic model which need more careful observations or aids them in strengthening this link between the microscopic phenomenon and the macroscopic outcomes presented to them. Formative assessment in the form of rich and timely feedback, along with the opportunity to revise responses, has been shown to support the development of learner self-regulation (Nicol & Macfarlane-Dick, 2006). Question prompts in learning environments can facilitate explanation construction, making justifications and monitoring and evaluation (Ge & Land, 2004).

- *Integrating micro-macro link*

There exists an integration of the micro-macro link when students summarize their complete understanding using multiple representations of the microscopic model, the macroscopic experimental evidence in the form of meter readings, and a graphical outcome. This aids in integrating the micro-macro link. Prompting learners to articulate their thinking helps them become more aware of what they know, which then makes their thinking available to them for reflection, monitoring, and revision (Scardamalia et al., 1989)

Overall, MIC-O-MAP follows the recommendations for a self-regulated environment by defining broad goals for learners in the form of learning objectives but a pre-defined path is not provided. MIC-O-MAP is a semi open-ended system where students set their sub-goals. Which feature or activity is to be accessed at what point of time in their learning curve is decided by the learner. Learners have the flexibility to alter their learning path, go back to a particular learning point, or simply restart their process. Thus, actions of monitoring and judgment are required of students when they interact with MIC-O-MAP. At each stage, learners are provided with scaffolds and prompts which aid in either making more careful observations or establishing a micro-macro link. This entire activity is carried out in the form of a dialogue with an agent so as to ease the follow-up of feedback.

MIC-O-MAP TEL environment has been developed and contains modules in topics in analog electronics. It runs on a browser and is compatible with most common browsers, screen sizes, and operating systems. The website hosting all modules of MIC-O-MAP can be found under Project TELoTS (http://www.et.iitb.ac.in/projects/telots/completedprojects.html). The modules can be directly downloaded by students and teachers. In addition, an instructional design template as well as the source code are available on the above webpage for researchers to build further new modules.

### Features and learning activities of MIC-O-MAP

The detailed features of MIC-O-MAP which aid in developing micro-macro thinking skills are explained below:

- *Simulation of the microscopic model*

Students are provided with a simulation of the microscopic model of a phenomenon and are asked to interact with it with the help of variable manipulation, text entry, and meter readings. It allows students to make observations in the microscopic worlds and later use these observations in order to establish a link. Features such as isolation and manipulation of parameters help students to develop an understanding of the relationships between physical concepts, variables, and phenomena (Rutten, van Joolingen & van der Veen, 2012).

- *Prediction questions*

Students are given a macroscopic situation and are asked to use the observations made by them in the microscopic world for predicting what might be the outcome of this situation. Learning material should provide opportunity for predicting the outcome of a situation/experiment (Gilbert, 2004).

- *Justification of prediction*

When students predict the outcome, they are asked to write an explanation on which their prediction is based. While doing this, they are asked to establish a link between the observations made by them in the microscopic world and its corresponding prediction in the macro-world. This is done as it is a good practice to students to have the opportunity to reason and adapt a known model to the specifications of a new problem (Wells et al., 1995).

- *Real-world answer for comparison and judgment*

To test their prediction, students are provided with the real-world macroscopic experimental outcome of the situation and are asked to decide if their prediction is correct. This is important because students should be able to analyze the outcomes of an experiment and be able to justify their conclusions (Wells et al., 1995).

- *Assertion and reasoning questions with customized feedback*

This activity begins with showing the students their own prediction and justification. They are then asked questions related to possible observations they made in the microscopic simulation that might have led them to answer in that manner. These questions aid in bringing the learners’ attention to key areas where observations need to be made. Prompts are also provided to help them link the micro- and macro-worlds, where feedback can be hidden and revived in accordance with the choice of the learner.

For example, if the student’s choice is a graph in which current varies linearly with voltage, they are asked questions such as: “Was there low electron flow initially? (micro-only question),” “Did the current increase only when you applied a certain amount of external voltage? (macro-only question),” “Was there a varying rise in the number of electrons when you vary the externally applied voltage? (micro-macro question).” If their answers are not consistent with the microscopic model, they are given feedback which helps them identify what was missed in their observations. They are asked to interact with the simulation again and to note that particular aspect in the simulation. These questions are also referred to as assertion and reasoning questions since we ask students to commit to one choice of answer and then engage them in a process of reasoning and inference. Students also monitor their learning while answering these questions; for example, when students are testing their prediction and ask for guidance, they are told to make more careful observations in order to establish a micro-macro link and asked if they would like to attempt this. If they give a positive answer, they are provided with a further set of questions which go deeper into the establishment of the micro-macro link. Making a decision of whether their recorded observations are sufficient for justifying their predictions or more scaffolding is needed is an instance of students being able to monitor one’s learning. This is a very crucial feature and is based on designing instruction using building blocks such as hints, scaffolds (“if confused”), and summarization (Wielinga et al., 1992).

**Fig. 4:**
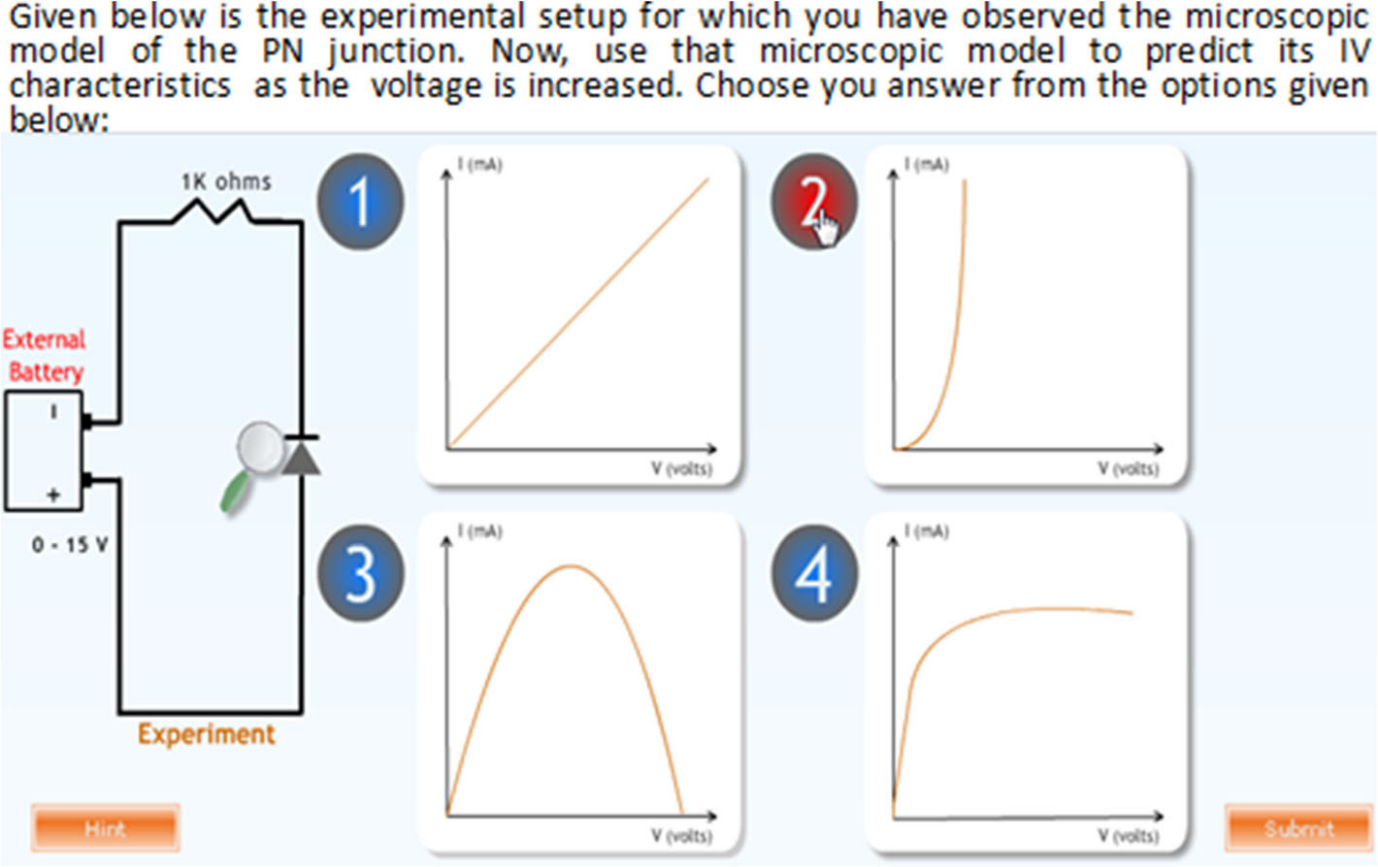
Prediction questions

**Fig. 5:**
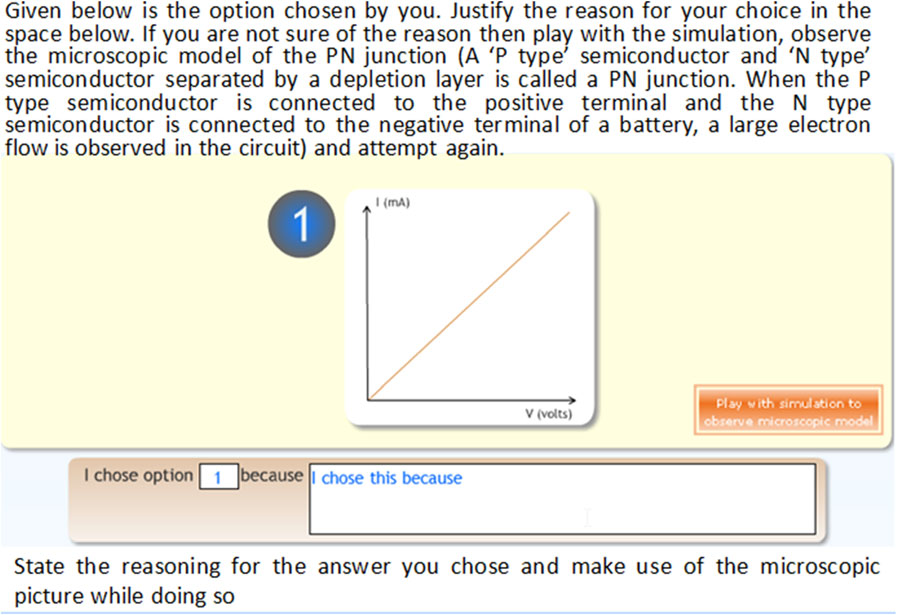
Justification of prediction

- *Multiple representations of microscopic process, macroscopic experiment, and graph*
  In order to summarize, students are shown the working of the microscopic model of the PN junction, the experimental setup with meter reading and a graphical outcome simultaneously (Figs. 4 and 5). This is needed in understanding the underlying concepts, relations, and processes (Kozma et al., 1997).

## Evaluation of MIC-O-MAP

The research questions addressed were as follows:

RQ1.1: Did students who worked with MIC-O-MAP develop micro-macro thinking skills?

RQ1.2: What are learning behaviors of different students as they interact with the various features and learning activities of MIC-O-MAP?

### Research design

RQ1.1 was answered using a two-group quasi-experimental research design to determine if there exists a difference in the performance (post-test scores) between the experimental group students who learn via MIC-O-MAP and the students in a control group. RQ1.2 was addressed using interaction analysis since the intention was to identify a productive learning path which can be used for interacting with this learning environment.

#### Pilot study

Before the main study, a pilot study was conducted to test the validity and usability of MIC-O-MAP and the validity of the post-test. A sample of three first year science/engineering major students worked with MIC-O-MAP and took the post-test. Results of the pilot study showed that students had no difficulty understanding the material in MIC-O-MAP or the post-test questions. The time required for the post-test (45 min) was used as a benchmark for the post-test time interval for the main study.

#### Participants

The participants were students from the first year undergraduate science and engineering programs from various colleges under Mumbai University, India. Formal invitations were sent to the departments of 12 colleges, and students were asked to formally register with us for the study. The topic being learned using the learning environment was PN junctions (forward biased) from the subject of physics. All these students had learned the domain knowledge present in the TEL environment in their college classes.

#### Procedure of the experiment

**Fig. 6:**
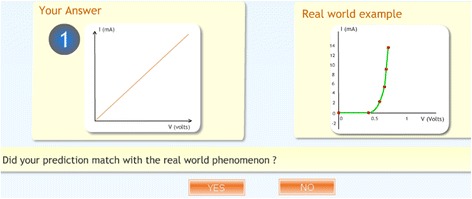
Real-world answer for comparison and judgment

**Fig. 7:**
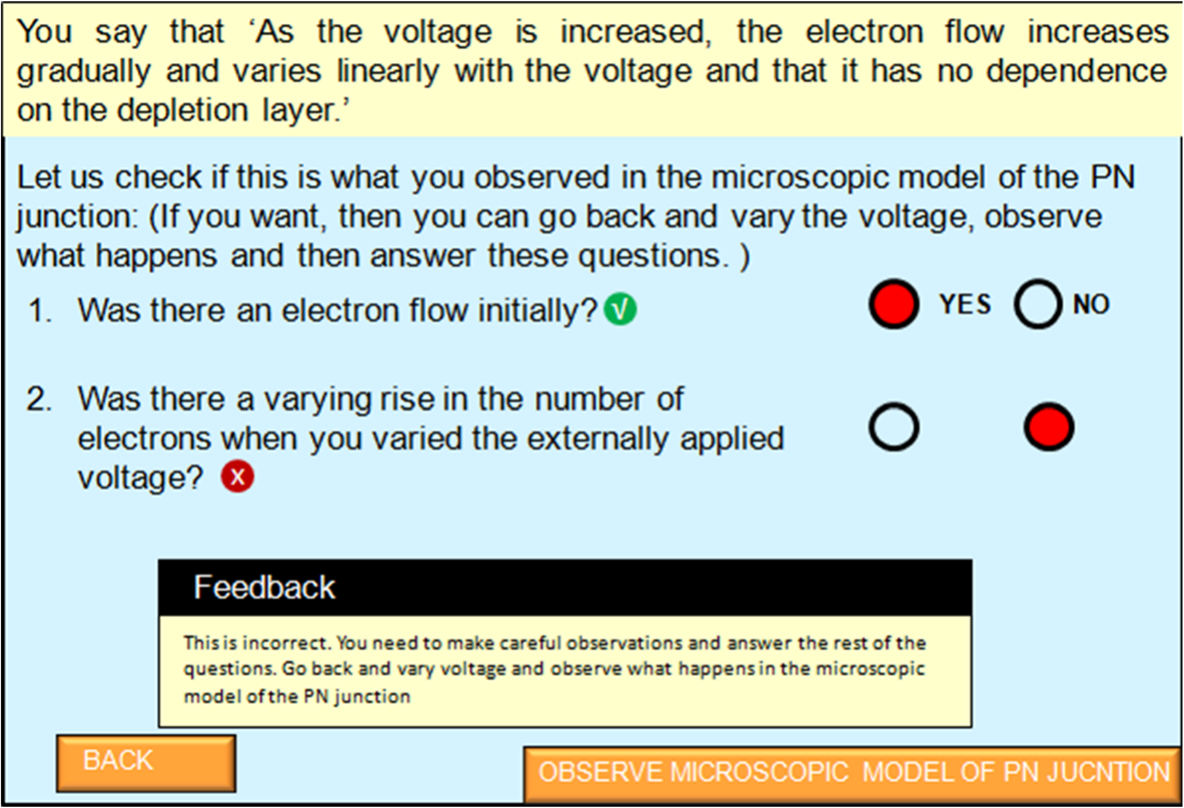
Assertion and reasoning questions with customized feedback

For RQ1.1, a quasi-experimental research design was adopted. The students (*N* = 73) who arrived at the experiment venue were assigned to two groups by randomized assignment. Group 1 (experimental group) contained 37 students while group 2 (control group) contained 36 students. To check if the experimental and control groups were equivalent in terms of prior knowledge, we compared their XII standard examination scores for the subject of physics. We did a *t*-test and found that there was no statistically significant difference between the groups, *t* (72) = 0.412, *p* = 0.685. Students in the experimental group were given the MIC-O-MAP TEL environment as the learning material. Students in the control group were given a visualization which contained the same simulation of the microscopic phenomenon as the TEL environment but did not contain the scaffolds and prompts which were present in MIC-O-MAP (Fig. 6). The control group students, in addition, were given a write-up containing a description of the macroscopic experiment and its microscopic explanation. This was done to ensure that the information contained in both the learning materials was equivalent. The main difference in the learning materials of the two groups was the features of question prompts for making predictions, writing explanation, conflict resolution questions, assertion and reasoning questions, and customized feedback (Fig. 7).

A total time period of 1 h was allotted to the students for learning the topic. At the end of the hour, a new physical phenomenon was presented to the student by means of a simulation depicting its microscopic explanation. Students were asked to interact with a simulation depicting the working of a PN junction diode in reverse biased conditions. Students were asked to answer post-test questions related to the new phenomenon.

For RQ1.2, in order to get an idea of the various student behaviors, purposive sampling was conducted to obtain 12 participants who scored high on the MIC-O-MAP post-test and 12 who scored low on the post-test. Post-test scores were used to identify high and low scorers: students in the top third were labeled as the “high-scorer” group and students at the bottom third were labeled as the “low-scorer” group.

#### Data sources and instruments

In order to answer RQ1.1 related to students’ development of micro-macro thinking skills, we administered a post-test which contained open-ended questions that mapped to the micro-macro thinking skill. These questions were given to five subject matter experts for content and construct validation. Each of these experts was given the micro-macro thinking skill test questions and was asked to mention if they were accurate and sufficient with respect to the content. The experts were asked to give us feedback if we have missed out any important area of the topic which needs to be covered. For the construct validity, the experts were also asked to mention if each question given in the test maps to the micro-macro thinking sub-skills of observing, explaining, predicting, and testing. They were requested to suggest alternate questions if the current questions were found to be inappropriate. This activity was conducted with each expert sequentially where we incorporated comments of one expert before giving to the next. This was repeated until saturation was obtained.

To grade the answers of the students to the post-test questions, rubrics have been adapted from the scientific abilities rubrics (Etkina et al., 2006). The rubrics contained descriptors of performance levels on criteria related to micro-macro thinking skill, such as being able to describe observations, make predictions, give explanations, and decide if experimental outcome and prediction agree. A sample rubric item related to prediction ability is shown in Table 1.

**Table 1:** Sample rubric item

Criterion	Score 0	Score 1	Score 2	Score 3
	Missing	Inadequate	Needs improvement	Target performance
Is able to make a reasonable prediction based on an explanation	No attempt is made to make a prediction	The prediction made does not follow from the explanation	A prediction is made that follows from the explanation but may contain minor errors or does not incorporate assumptions	A prediction is made that follows from the explanation and incorporates assumptions

For RQ1.2, while students studied the material, their screen activities were captured by My Screen Recorder software (http://www.deskshare.com/screen-recorder.aspx), screen-recording software. After this data collection, all these screen capturers were analyzed and later coded in accordance with the interaction pattern observed.

#### Data analysis technique

An inter-rater reliability analysis was performed to determine the consistency among raters who would be graded the post-tests. The inter-rater reliability for the raters was calculated using the statistic of Cohen’s kappa (*κ* = 0.839, *p* = 0.000). This indicates that there was a high agreement between the rubric scores allotted by the two raters. The Mann-Whitney *U* test was used to determine differences in the post-test scores on prediction-testing-revision abilities. This test is used to compare differences between two independent groups when the dependent variable is ordinal and not normally distributed. The test ranks the sample values from both sets of data. In order to analyze the qualitative data, an interaction analysis method (Jordan & Henderson, 1995) was used.

### Results

#### Post-test results

Table 2 shows the mean rubric scores of students’ post-test performance on criteria related to micro-macro thinking skill. The Mann-Whitney *U* test was performed to determine if the scores were significantly different.

**Table 2:** Mann-Whitney *U* test scores

Criteria	Experimental group mean, *N* = 37	Control group mean, *N* = 36	Mann-Whitney *U* test result
Describe observations without explanations	*M* = 1.97, *SD* = 0.644	*M* = 1.77, *SD* = 0.637	*U* = 562.00, *p* = 0.195
Devise an explanation for an observed pattern	*M* = 1.62, *SD* = 0.758	*M* = 0.83, *SD* = 0.845	*U* = 342.50, *p* = 0.000
Make prediction based on explanation	*M* = 1.62, *SD* = 0.794	*M* = 1.19, *SD* = 0.524	*U* = 437.00, *p* = 0.003
Decide whether the prediction and the experimental outcome agree	*M* = 2.27, *SD* = 1.017	*M* = 1.83, *SD* = 0.810	*U* = 455.00, *p* = 0.014
Revise the explanation when necessary	*M* = 0.64, *SD* = 0.856	*M* = 0.22, *SD* = 0.590	*U* = 475.50, *p* = 0.008

The results show that the experimental group showed a statistically significant difference at *p* = 0.05 level in their improvement in their abilities of making predictions, testing them, and then revising them as compared to the control group. There exists no statistically significant difference in their ability of making observations.

#### Interaction analysis

Learning with technology-based solutions is a student-centered active learning process entailing students’ self-propelled actions to acquire knowledge. It has been proposed to take into account the role of technologies in the interaction process (Finegold & Cooke, 2006; Roblyer & Wiencke, 2003). Learners’ familiarity with different functionalities of the interface increases their utilization, and each type of interaction depends on others in online learning environments (Sun & Hsu, 2013). Multiple methodologies have been reported useful for gaining insight into the interaction patterns of users while learning. Flanders Interaction Analysis Categories System (FIACS) is a classic system of interaction analysis used to study what is happening in a classroom when a teacher teaches (Amatari, 2015), whereas clickstream analysis is used for analyzing the record of screens or pages that user clicks on and sees, as they use a site or software product (Taniguchi, 2009). In intelligent tutoring systems, hidden Markov models have also been used to predict the interaction sequence for any given learning environment (Rychlik & Frendahl, 1993). In order to probe deeper into the manner in which students interact with MIC-O-MAP, it was important to use a methodology which investigates the navigation patterns of a user in a technology-based learning environment. It is reported in the literature to investigate the pattern of interaction of learners with a given environment to examine how students initiate, lead, or maintain interaction threads (Pawan et al., 2003; Yang & Wu, 2011).

**Fig. 8:**
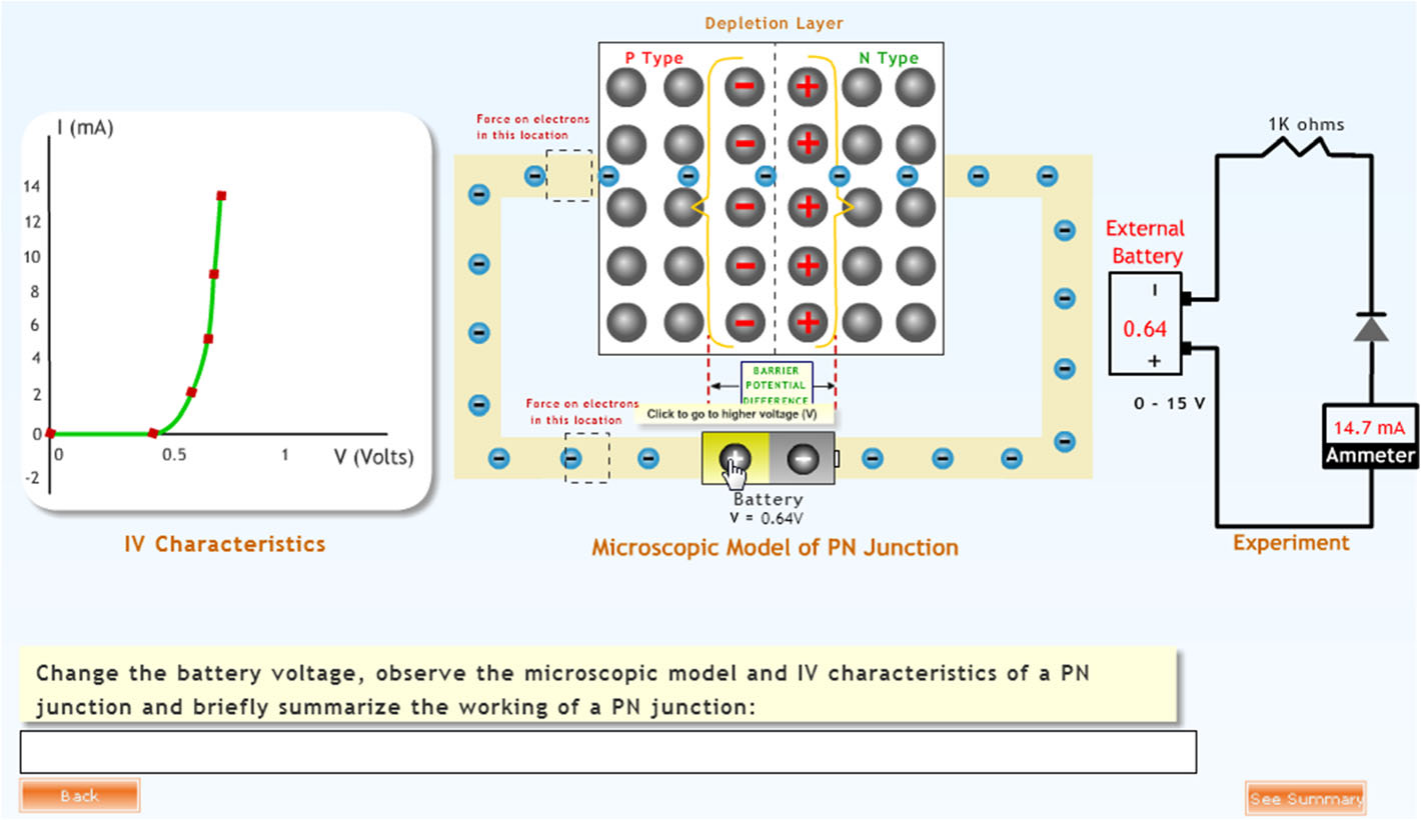
Multiple representations for summarization

Interaction analysis has been used to analyze video records for different purposes and on a large variety of topics (Jordan & Henderson, 1995). The entire screen activity of students was analyzed in accordance with the steps recommended in the above method. The structure of events provided a timeline for the activities we observed from a variety of perspectives, namely the total time spent by each student, the time spent on each activity, the percentage of time spent on each activity, the sequence of activities, and the responses given by high and low scorers to the feedback. Initially, we allocated codes for each line on-screen activity that was transcribed (Fig. 8). Later, we revised these codes and related them to each other in order to establish a behavior pattern for high-scoring versus low-scoring students (on the post-test) (Kenkre & Murthy, 2014).

**Fig. 9:**
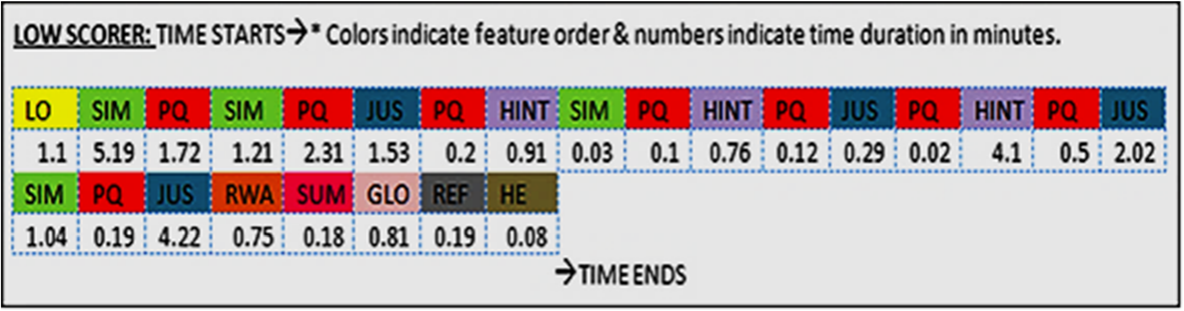
Learning path of a low scorer as he progresses in MIC-O-MAP

**Fig. 10:**
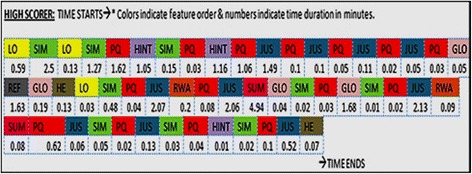
Learning path of a high scorer as she progresses in MIC-O-MAP

In order to generate a learning path, the on-screen activity was examined to record the features visited by every student. All features and learning activities of MIC-O-MAP visited by the learner are presented in the learning path in the order in which they were viewed. The time duration for which the learner was interacting with a specific feature is mentioned below the title of each feature. The learning path is unique for every student. Figures 9 and 10 depict a typical order in which a low scorer and a high scorer respectively interact with the features and learning activities in MIC-O-MAP.

The abbreviations in the figure refer to the various MIC-O-MAP features and learning activities: LO, learning objectives; SIM, interactive simulation of the microscopic model; PQ, prediction question; GLO, glossary; REF, references; HE, help; JUS, justification; RWA, experimental results for comparison; HINT, observation-based assertion and reasoning questions; ARQ, inference-based assertion and reasoning questions; SUM, multiple representations for summarization; and A, Assumptions.

On an average, all the students spent 29 (± 3.5) min interacting with the features and activities of MIC-O-MAP but they spent different time durations on different activities. To understand the possible reasons for the different times spent by different students, we compared the interaction behavior of students from the high- and low-scoring groups. We focused on the total number of times a certain activity was performed in a certain manner. For example, while writing a justification behind the prediction, high scorers made 26 attempts on an average to establish a micro-macro link whereas low scorers made 4 attempts to do the same. On the other hand, low scorers made 20 attempts in copying on-screen text and pasting it in the justification/notes whereas, in the high scorers, only 1 attempt to copy text was seen.

Figure 9 depicts the interaction pattern of a typical low-scoring student. This student began by interacting with the simulation of the microscopic model, but when confronted with a prediction-based question, he made a choice mostly by picking one of the options and tried to proceed to check if his answer was correct. In order to provide a justification for the prediction, he mostly copied the on-screen text and typed the same answer for multiple attempts. In case he went wrong, he directly proceeded to summarize. This text typed by him as justification was copied from either conceptual reasoning scaffolds or the glossary section.

Figure 10 indicates the interaction pattern of a typical high-scoring student. She initially made careful observations, then tried to answer the macroscopic prediction-based question. If unable to do so, she made use of the conceptual reasoning scaffolds to take note of which area of the microscopic model was to be viewed more carefully. The student then made an informed choice of prediction and tried multiple attempts at justifying it. Once a prediction was made, she tried to judge it in comparison with the experimental answer. If she was correct, she proceeded to the summarization activity wherein she had to establish a link between the microscopic phenomenon to its macroscopic outcome. In case her prediction was incorrect, she went to conceptual scaffolding-based questions and tried to improve their reasoning by making more careful observations or rephrasing their justification behind the prediction.

#### Productive value action

To further analyze the quality of learners’ interaction with MIC-O-MAP, we determined the productive value action (PVA). This is adapted from the term educationally valuable talk (EVT). EVT is defined as a particular interaction pattern in online discussion threads characterized as dialogic exchanges whereby a participant collaboratively displays construction, and at times, critical engagement with the ideas or key concepts that make up the topic of an online discussion, and builds knowledge through reasoning, articulations, creativity, and reflection (Bliss & Lawrence, 2009). A more useful distinction of “quality” can be made by determining whether a post is educationally valuable or educationally less valuable. The percent of educationally valuable talk was calculated by dividing the number of EVT posts by the total number of posts; thus, quality = the number of EVT posts / the total number of posts.

Adapting this definition, the quality of interaction in MIC-O-MAP was determined as follows: PVA = the number of productive attempts / the total number of attempts. Table 3 shows the PVA for learner actions in MIC-O-MAP.

**Table 3:** Productive value action (PVA) ratio for learners’ actions in MIC-O-MAP

Learner actions in MIC-O-MAP	PVA ratio
Attempts to establish a micro-macro link/no. of visits to justification writing	0.41
Making informed choice of prediction/no. of visits to prediction questions	0.08
Attempts to engage in sense-making/no. of visits to question prompts	0.58
Attempts to engage in sense-making/no. of visits to simulation of micro-world	0.227

### Discussion of DBR cycle 1

To answer RQ1.1, our findings of the two-group experiment showed that students who learn with MIC-O-MAP were able to develop observe-predict-test-revise skills and, in turn, the broader skill of micro-macro thinking. The results of the interaction analysis for RQ1.2 provided insights into a productive learning path undertaken by high-scoring students. However, in spite of this fact that students developed micro-macro thinking skills, we noticed that there were some problems when students interacted with this system. For example, even though high-scoring students made an informed choice of prediction, in total, they viewed the microscopic model 88 times, made a choice of prediction 132 times, but provided a justification with a micro-macro link only 62 times.

We analyzed possible problems learners faced as they interacted with MIC-O-MAP. Some of these problems were conceptual in nature; i.e., learners faced difficulty in establishing a micro-macro link, while others related to user interface issues. We then analyzed possible reasons for the problem, decided the most likely reason (based on experimental results and interaction analysis), and identified steps to redesign our intervention for the next iteration. Table 4 summarizes this.

**Table 4:** Problems from DBR cycle 1, possible reasons for their occurrence, and redesign steps

Problems seen in DBR cycle 1	Potential reason for problem	Proposed redesign to address problem
Learners struggle in establishing a micro-macro link, making an informed prediction and phrasing a justification	Disjoint activities—detailed question prompts and scaffolds, i.e., conceptual scaffolding questions are included in the revision phase only	Incorporating the question prompts at two stages: when a choice of graph is made and justification is written and when testing indicates incorrect prediction Option to take notes
While the number of observations made in the simulation of micro-world was high, the number of times feedback from the question prompts was followed was much lower	Overload on memory to recollect pointers from feedback while interacting with simulation of microscopic model	Simultaneous display of simulation of micro-world and other features including question prompts Including a pedagogical agent and providing feedback as a dialogue
Learners left the task half-way and stopped interacting after getting multiple answers incorrect	Perceiving question prompts as assessment	Including a pedagogical agent and providing feedback as a dialogue
Learners faced navigation difficulty while interacting with MIC-O-MAP interface	Features and learning activities are presented in a linear manner. In order to revisit an earlier activity, students had to visit all other activities. On-coming back/previous choices/answers were erased	Back tracing the path and retaining users’ action such as choices selected and text entry Scroll bars, reset, and back button and retention of answers/choices included

The implications and possible solutions are addressed below:

- A difficulty in establishing a micro-macro link is seen in being able to arrive at a correct prediction based on careful observations. A possible solution could be a simultaneous display of microscopic model, macroscopic prediction, and conceptual scaffolding prompts in order to ease establishing the linkage.
- Following instructions given in feedback is tedious. This tends to increase load on memory. Low scorers spend time in memorizing and do not reach the revision phase, some get answers correct, some misjudge, and some go back in choosing the correct answer and then proceed. They end up treating the conflict resolution as summative assessment and not formative. A possible solution can be altering the technique of giving feedback in the form of a dialogue with a pedagogical agent.
- The revision phase will be infused and merged into the main sequence. A note-taking section will be provided wherein students can write down important points which might need recall at a later point of time.

## Redesign of MIC-O-MAP: second cycle of DBR

### Refining the design features of MIC-O-MAP

An important feature we added in the revised version of MIC-O-MAP was a pedagogical agent, who acts like a mentor. The agent establishes a continuous dialogue with the learner and provides question prompts, guidance, and feedback as and when required. Pedagogical agents who act as tutors and mentors have been long used in intelligent and adaptive tutoring systems. Such agents have been used to assist students in establishing goals, monitoring emerging understanding, using effective strategies, and providing motivational scaffolding and personalized feedback (Azevedo, Cromley, & Seibert, 2004). Animated pedagogical agents have been recommended in the design of effective interactive multimedia to guide students’ attention, providing students with feedback and modeling, and guidance (Moreno, Mayer, Spires, & Lester, 2001).

We also included a note-taking feature into the environment so as to reduce the load on working memory and also serve a purpose to aid in strengthening the micro-macro links. Incorporating reflective activities is important to encourage an understanding-oriented approach. After completing their task, learners deliberately reflect on their experience to abstract the lessons learned and to consider how they performed in their self-directed learning (Hmelo et al., 2000). All the features of the environment are displayed simultaneously in order to ease out the process of sense-making.

**Fig. 11:**
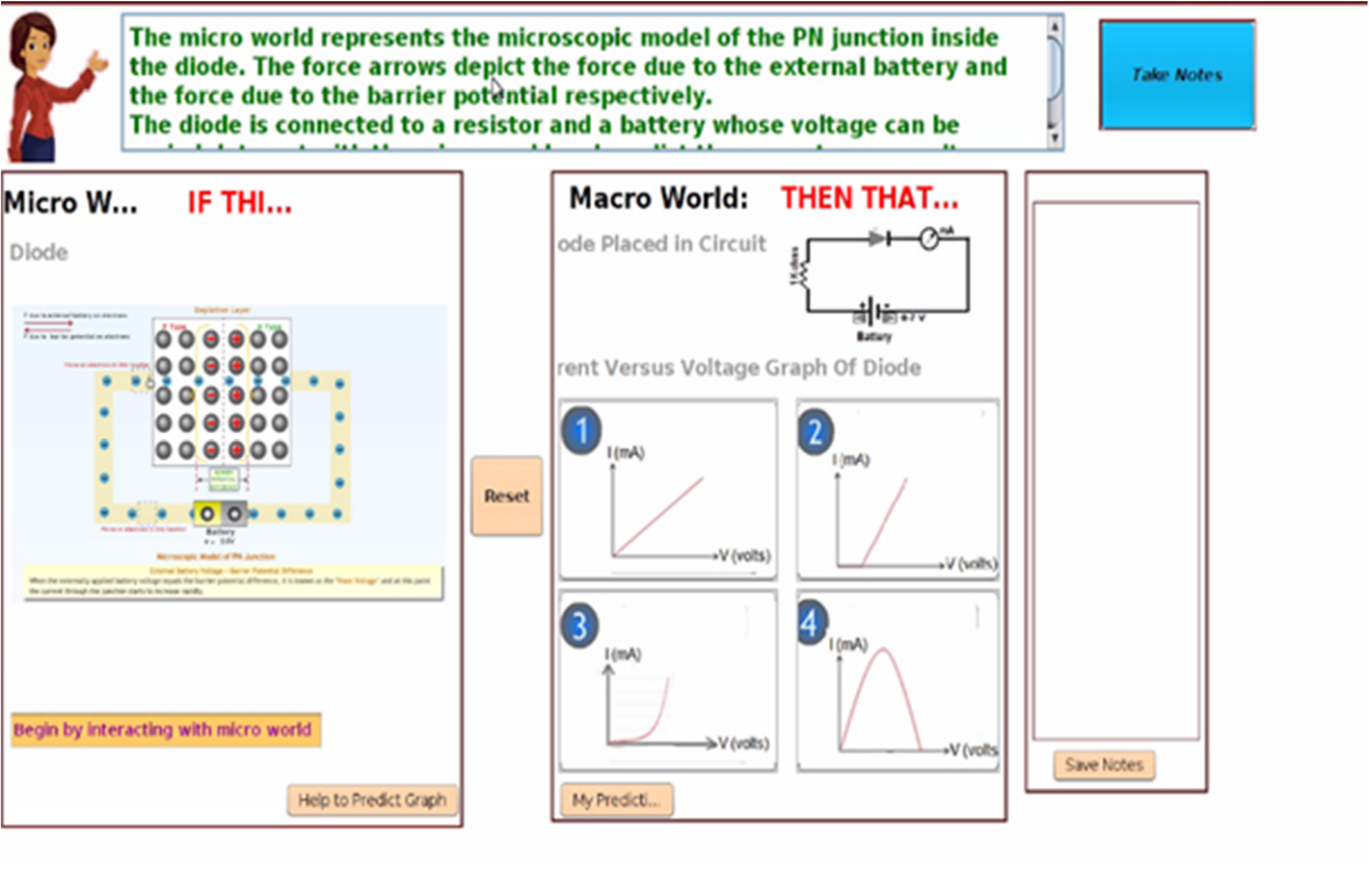
MIC-O-MAP with the pedagogical agent (left-hand side top), microscopic simulation and prediction question displayed simultaneously, and note taking (right-hand side section)

Path tracing and answer retention have been included into the system. Path tracing allows learners to decide or judge if they have achieved the broad goal set for them by the environment and the sub-goals set by themselves. Students can now retrace their path and improvise their answers or view their choices which strengthen the micro-macro link. The revised features of the environment are depicted in Fig. 11.

### Evaluation of revised MIC-O-MAP

#### Research design

This study consisted of a single group pre-post-test research design. It was used to answer the following research questions:

RQ2.1: Did students who worked with the revised version of MIC-O-MAP develop micro-macro thinking skills?

RQ2.2: How did the improvised design improve students’ learning path?

For RQ2.1, we chose a single group pre-post design instead of an experimental design, since the experimental study conducted in cycle 1 had already established that students who interacted with MIC-O-MAP learned micro-macro thinking skills more effectively than students in the control group who learned with simulation plus informative reading material (section “Results”). To investigate RQ2.2, we again conducted an interaction analysis as students interacted with MIC-O-MAP, similar to the study in cycle 1.

Participants were similar to the previous study, i.e., first year science and engineering students from various colleges affiliated to the University of Mumbai. The students (*N* = 46) were given MIC-O-MAP as the learning material. Before interacting with MIC-O-MAP, the physical phenomenon relevant to the MIC-O-MAP module (PN junction diode in reverse biased conditions) was presented to the student by means of a simulation (alone) depicting its microscopic explanation. They had to later answer pre-test questions related to this phenomenon. These pre-test questions tested students’ abilities of observe-predict-test-revise. Students then interacted with MIC-O-MAP for learning the topic of PN junctions in forward biased conditions. Hence, the microscopic model of the same material (PN junction) was altered on account of its biasing conditions and students had to make sense of the altered microscopic model and predict its macroscopic experimental outcome. Students worked for 1 h with the learning material in MIC-O-MAP. They then answered a post-test similar to the pre-test. The post-test questions and rubrics used for data analysis were the same as those used in cycle 1 (section “Research design”).

In the revised version of MIC-O-MAP, the revision phase has been merged into the main phase. Initially, when students interact with the simulation of the microscopic world, they are provided with the sheet of questions until the question asking them to justify the prediction. Here, they are developing the skill of micro-macro thinking while learning and attempting the prediction question. Once they completed answering this, they are provided with another sheet in which the real-world answer is given for testing and judgment. Here, students are also provided with another blank sheet in which they can elaborate or rewrite the explanation for the prediction wherein students are revising their previous answers. While answering the post-test questions, the answers written by students are given a rubric-based score for each sub-skill of observe, explain, predict, and test. There is no question explicitly demarcating a revision of student answers corresponding to these skills. For the first version of MIC-O-MAP, we had included the last question titled “revision” and students were asked to answer it after testing their prediction. For the second version of MIC-O-MAP, the answer written on the blank sheet provided with the real-world answer is absorbed into the second question where students are asked to devise an explanation for an observed pattern. Due to this, the post-test for the first version had five questions whereas the post-test for the revised version of MIC-O-MAP had four questions mapped to each sub-skill.

The Wilcoxon signed-rank test was used to determine differences in the pre- and post-test scores on observation-prediction-testing-explanation abilities. It is the nonparametric test equivalent to the dependent *t*-test. It is used to compare two sets of scores that come from the same participants.

Interaction analysis was carried out on a sub-set of the total number of students who answered the post-test questions in cycle 2. For this analysis, a *purposive sampling technique was used in order to choose the students who received high scores (*N* = 10) on the micro-macro thinking skills test. The chosen students were high-scoring students, and hence, an analysis of their interaction with MIC-O-MAP would provide insights about their learning paths. These students were chosen since they indicated the development of micro-macro thinking skills. We compared their screen capture recordings with those of the high-scoring students from cycle 1. This would help in contrasting the two in order to confirm that the learning challenges being faced by students have been addressed by the refined design of MIC-O-MAP.

#### Results of learning with MIC-O-MAP from cycle 2

The average learning time was 43.9 min. The inter-rater reliability for the raters was calculated using the statistic of Cohen’s kappa (*κ* = 0.839, *p* = 0.000). This indicates that there was a high agreement between the rubric scores allotted by the two raters. Table 5 shows the mean rubric scores of students’ pre- and post-test performance.

**Table 5:** The Wilcoxon signed-rank test results

Criteria	Pre-test mean, *N* = 46	Post-test mean, *N* = 46	Wilcoxon signed-rank test results
Describe observations without explanations	*M* = 1.27, *SD* = 0.962	*M* = 1.84, *SD* = 0.851	*Z* = − 3.953, *p* = 0.000
Devise an explanation for an observed pattern	*M* = 1.48, *SD* = 1.12	*M* = 2.13, *SD* = 0.694	*Z* = − 3.018, *p* = 0.003
Make prediction based on explanation	*M* = 1.75, *SD* = 0.829	*M* = 2.53, *SD* = 0.694	*Z* = − 4.284, *p* = 0.000
Decide whether the prediction and the experimental outcome agree	*M* = 2.42, *SD* = 1.177	*M* = 3.0, *SD* = 0.000	*Z* = − 2.887, *p* = 0.004

The results showed a statistically significant difference between pre- and post-test scores at *p* < 0.001 level in students’ abilities of making observations, predictions, and explanations and testing them. Students were already familiar with the content prior to the pre-test via the simulation (alone), yet they did not score high on pre-test. Since the post-test scores were statistically significantly higher, we infer that the MIC-O-MAP intervention (between the pre- and post-tests) had an effect on students’ learning.

#### Interaction analysis with MIC-O-MAP from cycle 2

**Fig. 12:**
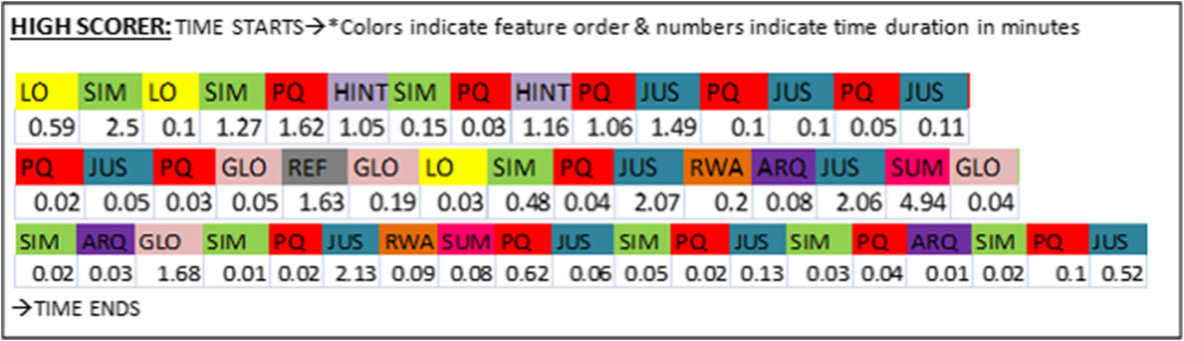
Learning path of students from cycle 2

A qualitative analysis was done similar to the study in cycle 1, and the same codes were allocated after viewing the screen recordings (section “Data sources and instruments”). Figure 12 depicts the learning path adopted by a student, which is typical of the path of all students in this study. This path is similar to that of high scorers from cycle 1 (Fig. 10).

To show that the effect of the redesigned version of MIC-O-MAP, we calculated the PVAs related to the problems in the original version of MIC-O-MAP. Table 6 shows the average PVA ratios for ten high-scoring students in cycle 2 and compares it with the results from cycle 1 (values from Table 3).

**Table 6:** Productive value action of students interacting with revised MIC-O-MAP

Action	PVA ratio of learners in cycle 2	PVA ratio of learners in cycle 1 (from Table 3)	Improvements of learner interactions with revised MIC-O-MAP
Attempting to establish a micro-macro link in justification	0.8	0.41	Learners make more successful attempts of establishing micro-macro link and show more instances of making an informed prediction based on a justification
Engage in sense-making by answering question prompts	1.09	0.58	Fewer instances of learners quitting MIC-O-MAP activities half-way
Engage in sense-making by careful observation of simulation	0.54	0.227	More instances of learner acting on feedback provided on their responses

The improved PVA ratios in the revised version of MIC-O-MAP from cycle 2 indicate that the desired action is being performed by the student while interacting with the learning environment, thus addressing the problems detected in the original version. There exist a higher number of attempts in establishing a micro-macro link and in sense-making with question prompts and observations of the simulation. The navigation difficulty has comparatively eased out due to enabling path tracing and retention of the choices and text entered by the student.

## Overall discussion

Overall, we find that students learning with MIC-O-MAP develop micro-macro thinking skills, as an answer to RQ1.1: “Did students who worked with MIC-O-MAP develop micro-macro thinking skills?” has been found by conducting quantitative studies. For the first version of MIC-O-MAP, a two-group quasi-experimental research design was adopted and it was found that students learning with MIC-O-MAP secure higher scores on a post-test on micro-macro thinking skills applied to a new topic than the students who learn with traditional visualizations which do not have the features and learning activities included in MIC-O-MAP. The difference between these two groups of students was found to be statistically significant.

For answering RQ1.2: “What are learning behaviours of different students as they interact with the various features and learning activities of MIC-O-MAP?,” interaction analysis was carried out. The learning paths and frequency of visit for a MIC-O-MAP feature based on a learning purpose (reported as productive value actions) have been analyzed. It was found that there exist differences in the learning paths of high-scoring and low-scoring students. High-scoring students are found to navigate through MIC-O-MAP by going back and forth through all of the features based on a learning goal, which is further mapped to a micro-macro thinking sub-skill (such as prediction). On the other hand, low-scoring students are found to follow a chronological order of interacting with each feature till they complete the interaction with the entire module. We have also reported the learning difficulties faced by students while working with the first version of MIC-O-MAP.

Following this, in order to answer RQ2.1: “Did students who worked with the revised version of MIC-O-MAP develop micro-macro thinking skills?” for the second version of MIC-O-MAP, a pre-post-test research design was adopted. The post-test scores were found to be statistically significantly higher than the pre-test scores for the topic which was tested. The limitation of the above findings is that the test has been conducted immediately after the students have interacted with MIC-O-MAP, due to which application of macro-micro thinking skill after some span of time has not been evaluated. Another limitation is that the topics that have been used as the subject content contain direct mappings between microscopic and macroscopic variables (for example, electron motion with current). Further examination will be required for topics with more complexity, such as when multiple and interacting microscopic processes together lead to a macroscopic behavior.

With respect to RQ2.2, students were found to go back and forth between all relevant features and activities of MIC-O-MAP at a higher rate than what was observed in cycle 1. We also found that students were continuously engaged in the process of scientific argumentation—either while justifying their predictions or for revising their prediction after comparison with experimental outcomes. In addition, the improvisation undertaken in the revised design of MIC-O-MAP reduced the interaction-based problems which had arisen in the original design. In the revised MIC-O-MAP, students were able to achieve the goals set for themselves in order to develop each skill in fewer attempts. While making observations in the microscopic world, they use the simulation of the microscopic picture and question prompts as scaffolds in order to identify key areas which needed attention, for example the knee voltage region of a voltage-current graph of a diode, where rapid changes in current occur. Students monitored their learning through a dialogue with the pedagogical agent. This erased the look and feel of being tested; instead, it provided students a feel of being mentored and supported when necessary. There was stronger evidence of the establishment of micro-macro links, since students used question prompts as pointers towards making more careful observations, also for sense-making between the unseen dynamics and the visible manipulative outcomes. At all times, students made their own choices about which combination of features and learning activities to interact with at each point in their learning route. This is crucial in a self-regulated learning environment.

Changes in the user interaction design led to improvement in usability, which, in turn, led to more focus on the relevant learning tasks. For example, allowing the learner to trace his/her path helped in the ease of navigation as students did not need to restart each time they wished to go back to a certain area of interaction. Retention of their previous responses and choices helped bring the focus on improvisation of their answers instead of rewriting them for the sake of moving back and forth. The note-taking option that was provided in the revised MIC-O-MAP helped ease cognitive load on working memory; thus, students could instead concentrate on the learning task at hand. The interaction analysis showed that students use the features of MIC-O-MAP and do the learning activities for taking stock of the areas which need further careful observations, planning, and monitoring their approach in order to establish a micro-macro link and to quickly as well as correctly reach the task which needs more rework.

A limitation of this study is that it is currently developed in only a few topics within the domain of basic electronics. One direction of our future work is to create and test multiple modules in topics within the scope to generalize these findings. This scope is restricted to topics which have a real-world tangible element that can be tested in a laboratory, and can be explained by a process at the microscopic level in terms of the objects present and interactions between them. Another limitation is related to the sample size and nature of the participants. While there were statistically significant differences, the sample size in our studies was only medium (50–100) and needs to be tested with larger numbers. Our studies were restricted to urban colleges where students have proficiency in English and are familiar with learning on their own from a computer-based learning environment. Thus, our study can be generalized to students in similar settings. However, in contexts where students are not comfortable with self-learning, MIC-O-MAP may require support from instructors trained in inquiry practices as well as in teaching with ICT-based materials. From the methodological perspective, we conducted the interaction analysis manually. While we took care of reliability issues, the manual process was effort-intensive and is not scalable. An improvement would be to include either partially or fully automated educational data mining or learning analytics (for example, Ferguson, 2012).

## Summary and conclusion

In this paper, we reported on two design-based research cycles of design, development, and evaluation to develop a TEL environment, MIC-O-MAP, addressing micro-macro thinking skills for undergraduate science and engineering students. This DBR approach helped us identify features and learning activities in MIC-O-MAP that led to productive learning, such as multi-level-linked representations and conflict resolution questions with customized feedback which aid students in establishing a link between the microscopic and macroscopic worlds. Findings from the quantitative studies helped confirm that learning with MIC-O-MAP aids in the development of micro-macro thinking skills whereas the qualitative studies gave rise to specific understanding of how learner interaction with TEL environments can support this development. The qualitative studies also gave rise to recommendations while designing such TEL environments, for example the role of the pedagogical agent to ease assessment anxiety related to question prompts and instead transform it into a mentoring process. The iterative and closely linked process involving the design (and redesign) of MIC-O-MAP and its evaluation formed the backbone of our research process. It helped us arrive at an effective TEL environment customized to our goals and also helped us identify aspects of a local instructional theory (Cobb et al., 2003). Towards this, the detailed qualitative interaction analysis was invaluable, and hence, we recommend such an analysis to be part of the evaluation of any TEL environment designed by researchers.

In terms of future work, we plan to use the design features and learning activities identified from this study to create further MIC-O-MAP modules in relevant topics. Insights from our study can be used in the technological as well as pedagogical design of effective TEL environments. Also, the learning path of high scorers indicates a productive strategy to interact and learn from MIC-O-MAP. We propose to use these productive strategies to make recommendations for teachers as well as students in order to gain most benefit from TEL environments such as MIC-O-MAP.
